# Evaluation of Food Trucks' Hygiene During Mass Gathering Events

**DOI:** 10.7759/cureus.19470

**Published:** 2021-11-11

**Authors:** Omar B Ahmed, Osama A Attalah, Hamza Assaggaf

**Affiliations:** 1 Environmental and Health Research, Umm Al-Qura University, Makkah, SAU; 2 Food Safety, Umm Al-Qura University, Makkah, SAU; 3 Laboratory Medicine, Umm Al-Qura University, Makkah, SAU

**Keywords:** mass gathering, mesophilic, temperature, psychotropic, food trucks, hygiene

## Abstract

During any event, food- and waterborne diseases represent a health risk, so food should be kept in a chilled state while being supplied in the trucks before distribution to consumers. The study aimed to evaluate temperature conditions, microbial contamination of food trucks, and their compliance to the basic local and international standards of food safety during a mass gathering event (pilgrimage). Fifty food trucks were evaluated for proper food storing and microbial contamination (load). A food truck inspection checklist was made to check if the trucks followed the standards of food safety and hygiene. The results showed that 90% of the trucks’ refrigerators were between 3 and 5°C. The number of total mesophilic and psychrotrophic bacteria in the contact surfaces varied between 1.93-6.16 (mean 4.0433) and 0.08-4.50 (mean 3.3340) log CFU (colony-forming units)/100 cm^2^, respectively. The studied food trucks met the standards of food safety and hygiene with a low mean level (45.2%), which was seen in monitoring thermometers/cold chain loggers, proper record of activities, proper disposal of food product waste, and proper practice of handwashing techniques. There were significant associations (p-value < 0.05) found between temperature readings and mesophilic microbial load. Efforts should be increased to reduce food loss by continuous monitoring of these trucks during mass gathering events.

## Introduction

Makkah city (Western region of Kingdom of Saudi Arabia (KSA)) is one of the most crowded cities during mass gatherings (pilgrimage) in the world; hence, food and drinks products should be properly transported, handled, and stored. Generally, transportation of food products between KSA regions is done by trucks equipped with refrigerators. These trucks or vehicles loaded with food products should meet certain standards of food safety and hygiene. The main requirements of these standards are the temperature monitoring to keep optimum storage, employee hygiene, provision of equipment, and cleaning/sanitary conditions [1,2]. Temperature is an important factor that inﬂuences the safety of food products and plays an important role in the growth of microorganisms [3]. The storage of food at refrigeration temperature will inhibit the growth of psychrotrophic bacteria and delay the spoilage of food products. Because these bacteria, either Gram-positive or Gram-negative, are able to grow at cool temperatures and have the ability to produce heat-stable hydrolytic enzymes and may cause the spoilage of the heat-treated food products (e.g. dairy) as the result of post contamination of these products [4], the food should be kept in a chilled state while being supplied. When the food is not kept in the desired temperature range, there may be a growth of microorganisms in the food product, rendering it unhealthy and result in an increased amount of food product waste [5]. It was estimated that 48 million Americans suffer from domestically acquired foodborne illness associated with 31 identified pathogens and a broad category of unspecified agents [6]. In addition, pathogens can cause 10 million foodborne illnesses and may cost more than 50 billion dollars in the United States; it may lead to about 120,000 hospitalizations resulting in 3,000 deaths and 128,000 hospitalizations [6,7]. Temperature loggers are growing in popularity to control ambient conditions, particularly for the storage of food products. These loggers have distinguished features such as continuous temperature monitoring, detailed time-stamped temperature logs, temperature excursion indicator (visual or via alerts), and reliable storage and transfer of temperature data [8]. It was reported that the use of temperature loggers would reduce the annually wasted food by more than 14% of the consumption [9]. Also, food refrigeration needs to be applied throughout the entire cold chain within the food industry to ensure food safety. The study aimed to evaluate food ambient conditions (storage temperature), microbial contamination (load), and the compliance of food trucks to the basic standards of food safety during a mass gathering event (pilgrimage) in Makkah city in the western region of KSA.

## Materials and methods

The study was conducted during August (2018) by monitoring the temperature and microbial load of 50 refrigerated food transport trucks coming from different regions of KSA and destined to Makkah city. The selected food trucks were packed with bottled water, bottles of refreshments, juices, milk, loaves of bread, and pastries. The trucks were also evaluated for their compliance to food safety and hygiene standards.

Monitoring of storage temperature

**Figure 1:**
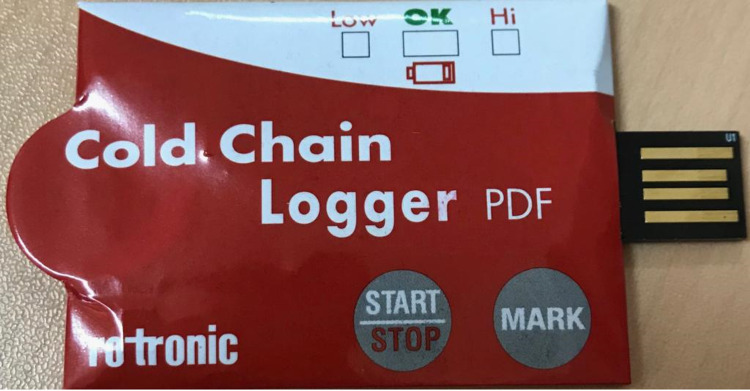
The type of cold chain logger used in the study.

The field test was designed and conducted successfully with 50 data loggers inserted in 50 transport trucks randomly distributed in three places (Arafa, Muzadlifa, Mina), which started loading in the period 17-21 August 2018 (6-9/12/1439 H). The test was performed by inserting data loggers (TL-CC1 Cold Chain logger, ROTRONIC AG Switzerland) (Figure 1) at the contact surfaces of walls of food trucks. The logger was then lifted at the end of 21st of August 2018, which corresponded to 9/12/1439 H (Arafa day), and the unloading data and temperature curves were prepared according to the manufacturer's instructions and specifications.

Microbiological evaluation (microbial load)

Swab samples from internal surfaces of the trucks, which were moistened with buffered peptone water, were obtained from the interior sides of each truck by rotating on the internal surface with an area of 100 cm^2^ from each truck. The swabs were transported back to the laboratory under cold conditions (4 ± 1.0°C) and processed within 1 h. Each bag containing swabs was diluted into 1/10 with sterile saline and shaken for 2 min. After serial dilution, 1 ml of the suspension from each dilution was inoculated to plate count agar (for 48 h at 37°C) and plate count agar (10 days at 6.5 ± 0.5°C) to examine the total count of mesophilic and psychrotrophic bacteria, respectively [10]. Each microbiological test was performed in duplicate.

Inspection checklist

A food truck inspection checklist was made to check if the trucks followed the standards of food safety and hygiene. The checklist included permission, safety and storage, employee hygiene, equipment, and cleaning/sanitary conditions.

Statistical analysis

Statistical analysis was used to study the associations (chi-squared test) and correlations (Pearson's correlation) between the temperatures and microbial load. The study variable, a p-value of <0.05, was considered as statistically significant.

## Results

**Figure 2:**
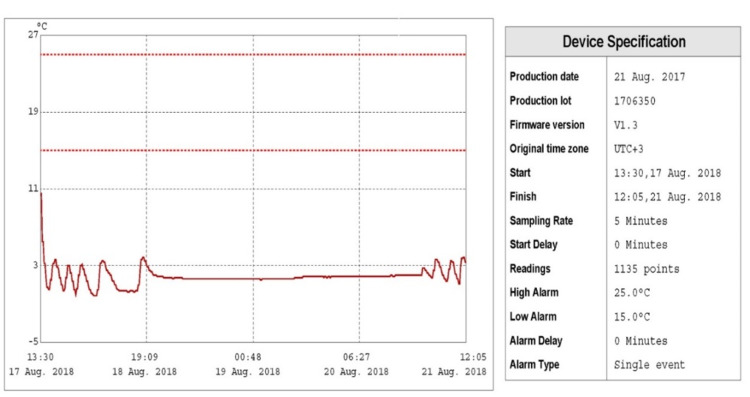
Normal temperature proﬁle of food storage along the cold chain.

**Figure 3:**
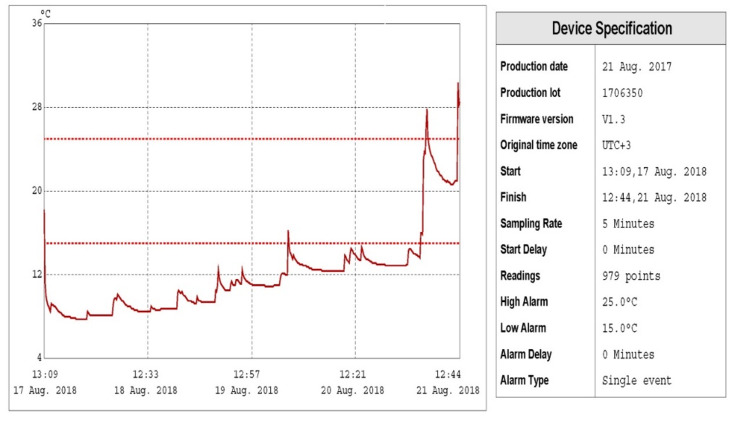
Variability of temperature proﬁle of food storage along the cold chain.

To evaluate the health standards of these food trucks, field testing and microbial load testing were designed and conducted in 50 transport trucks. Table 1 showed that in 90% (45) of the trucks’ refrigerators tested, the temperatures within the trucks were between 3 and 17°C with a mean of 4.56°C from day 17 to 21 August 2018 (Figure 2). Table 1 also shows that the number of total mesophilic and psychrotrophic bacteria in the internal contact surfaces of food trucks was 1.93-6.16 and 0.08-4.50 log CFU (colony-forming units)/100 cm^2^, respectively (Figure 3). The mean value of total number of mesophilic bacteria and psychrotrophic bacteria in the internal contact surfaces of food trucks was 4.0433 and 3.3340 log CFU/100 cm^2^, respectively.

**Table 1:** Evaluation of food truck storage temperatures and microbial contamination. CFU: colony-forming unit.

Reading	Min.	Max.	Mean	Deviation	Correlation	p-Value significance (2-tailed)
Mesophilic bacterial count log "CFU/100 cm^2^"	1.93	6.16	4.0433	0.59010	0.503	0.000
Psychrotrophic bacterial count log "CFU/100 cm^2^"	0.08	4.50	3.3340	1.15291	-0.096	0.507
Temperature (°C)	3.0	17.0	4.56	2.92358	-	-

Results show that there was a significant correlation between temperature values and total mesophilic bacterial count (p < 0.05), but no significant correlation between temperature values and total psychrotrophic count (p > 0.05) to be concluded (Table 1). The present study showed that the recorded temperatures in 10% (5) of trucks during the distribution stage on 21 August 2018 showed greater variability with broader temperature ranges, where fluctuations in temperatures reached more than 10°C (Figure 3). Table 2 shows that food trucks met the standards of food safety and hygiene with a low mean level (45.2%); in addition, it shows that all trucks, 100% (50), have neither thermometers/cold chain loggers nor sinks for hand-washing, while 94% (47) of them lack proper record of activities such as contents and cleaning schedules. Statistically, there were significant associations (p < 0.05) between temperature readings and some of the variables for food safety and hygiene standards such as proper record of activities in the trucks, proper food product waste disposal, and outside look (cleaning) of truck.

**Table 2:** Results of inspection checklist. F = frequency.

Checklist	Item	Yes % (F)	No % (F)	Chi squared test (p-value)
Permission	Licenses and permits	88 (44)	12 (6)	0.234
	Proper record of activities (contents, cleaning schedules)	6 (3)	94 (47)	0.013
Safety and storage	All food and drinks are proven safe for consumption	30 (15)	70 (35)	0.369
	Food and drinks storage containers are properly labeled	90 (45)	10 (5)	0.143
	Food products are not left out on the truck	30 (15)	70 (35)	0.698
	All food and drinks are stored at safe temperatures	40 (20)	60 (30)	0.135
Employee hygiene	Employees practice proper hand-washing techniques	10 (5)	90 (45)	0.214
	Employees are up-to-date on food safety practices	16 (8)	84 (42)	0.990
	Employees are healthy with protective wear	26 (13)	74 (37)	0.291
Equipment	Availability of thermometers or cold chain loggers	0 (0)	100 (50)	NA
	Cleaning materials are kept in a designated place	90 (45)	10 (5)	0.106
	Sink for hand-washing is available	0 (0)	100 (50)	NA
	Adequate ventilation and lighting	80 (40)	20 (10)	0.613
	Air curtains are available	30 (15)	70 (35)	0.631
Cleaning and sanitary conditions	All surfaces are clean and in good condition	90 (45)	10 (5)	0.153
	Food product wastes are properly disposed	10 (5)	90 (45)	0.004
	Outside of truck is clean and in good condition	88 (44)	12 (6)	0.000
	No signs of flies and pests	90 (45)	10 (5)	0.153
Mean		45.2%	54.8%	

## Discussion

In order to provide an effective storage life during Hajj (pilgrimage), the foods need to be stored at least substantially within 2-6°C. These low temperatures are needed to preserve the flavor, odor, color, and texture of the food by retarding chemical changes, the action of food enzymes, and by eliminating the growth of microorganisms capable of growth near to this range. Food trucks loaded with numerous containers of bottled water and meals are distributed everywhere in KSA regions, particularly in Makkah, during Hajj season when millions of people come together in a specific small location for doing Hajj rituals. Temperature data recorded during the study period since the start of transporting the food products from different regions of the KSA to Makkah city were found quite consistent with temperatures ranging mainly from 3 to 5°C. Our results are similar to the mean temperature of the complete cold chain of food products in Europe which was found between 3.5 and 5°C [11]. Some fluctuations were observed throughout the cold chain stages; however, differences between the different cold chain stages were also observed. Also, the study showed that the number of total mesophilic bacteria in the internal contact surfaces of food trucks was 1.93-6.16 log CFU/100 cm^2^ with a mean value of 4.0433 log CFU/100 cm^2^ while the total number of psychrotrophic bacteria was 0.08-4.50 log CFU/100 cm^2^ with a mean value of 3.3340 log CFU/100 cm^2^. Psychrophilic microorganisms have been described as microorganisms capable of appreciable growth at refrigeration temperatures. Such microorganisms can grow at temperatures lower than 15°C. One study [12] found deficient cleanliness in 55% of refrigerators with a mean total number of psychrotrophic bacteria being 82.3 CFU/m^3^. Similar observations were seen where the bacterial count changed significantly with effect of temperature [13-15]. Monitoring temperature when storing or transporting food would prevent foodborne illness and may avoid potentially severe health hazards during mass gatherings. Temperature is one of the major controlling factors of food quality and food safety because of its influence on microbial growth rates. Despite the fact that low temperature can reduce the growth rate of many species of microorganisms, it has been reported that psychrotrophic microorganisms can grow at normal refrigeration temperatures [16]. In cooler conditions, psychrotrophic bacteria constitute a higher percentage of the microflora than in warmer conditions [17,18]. Results showed that there was a significant correlation between temperature values and the total mesophilic bacterial count (p < 0.05). Therefore, temperature values may be used as an indicator of the total bacterial count in the food trucks. The finding of this study is similar to those of previous studies [13,14]. The variability in the recorded temperature profiles can be mainly attributed to the excessive times of opening doors of the trucks while distributing food to consumers. The effects of door openings were studied previously in refrigerated cargo transport [19,20]. In general, as the temperature is lowered, fewer organisms are capable of growth and the rate of multiplication of these organisms becomes progressively slower. The studied food trucks met a low mean level of the standards of food safety and hygiene, in addition to all trucks (100%) having neither thermometers/cold chain loggers nor sinks for hand-washing, and most of them (94%) lack proper record of activities such as contents and cleaning schedules, and 90% have neither proper disposal of food product waste nor proper practice of hand-washing techniques. In addition, the study showed that most of the truck employees (84%) were not up-to-date on food safety practices, and 70% of the trucks lacked air curtains. Fortunately, the study showed that most of the trucks (90%) met proper standards of labeling of food and drinks storage containers and safe storing of cleaning materials; their floors, sinks, and walls were clean and in good condition and free of flies and pests. Moreover, 88% of them were licensed and had permits and looked clean in appearance. Worldwide, refrigeration has been considered an important food safety factor in reducing food loss, waste, and foodborne illness in most countries [21]. Similar studies showed that temperature monitoring and control were essential in food trucks working [22,23] because they were necessary for maintaining food safety and quality. Appropriate storage with protection from weather, microbes, and pests can keep food safe and reduce food loss and waste. Many studies showed expressive temperature raises in the absence of air curtains [19,20,22]. There was significant association (p < 0.05) between temperature readings and proper record of activities in the trucks, proper food product waste disposal, and outside look (cleaning) of truck.

## Conclusions

The hygiene evaluation of the studied food trucks showed a variability in temperature and microbial profiles due to to the excessive times of opening doors of the trucks while distributing food to consumers. The low mean level of the standards was seen in monitoring thermometers/cold chain loggers, proper record of activities, proper disposal of food product waste, and proper practice of hand-washing techniques. There were significant associations (p < 0.05) found between temperature readings and mesophilic microbial load. Efforts should be increased to reduce food loss by continuous monitoring of these trucks during pilgrimage events.

## References

[1] Ackerley N, Sertkaya A, Lange R (2010). Food transportation safety: characterizing risks and controls by use of expert opinion. Food Prot Trends.

[2] Esfarjani F, Khoshtinat K, Zargaraan A (2019). Evaluating the rancidity and quality of discarded oils in fast food restaurants. Food Sci Nutr.

[3] Waqas M, Almeelbi T, Nizami AS (2018). Resource recovery of food waste through continuous thermophilic in-vessel composting. Environ Sci Pollut Res Int.

[4] Samardžija D, Zamberlin Š, Pogačić T (2012). Psychrotrophic bacteria and milk and dairy products quality. Mljekarstvo.

[5] Leistner L (2000). Basic aspects of food preservation by hurdle technology. Int J Food Microbiol.

[6] Scharff RL (2012). Economic burden from health losses due to foodborne illness in the United States. J Food Prot.

[7] Scallan E, Hoekstra RM, Angulo FJ (2011). Foodborne illness acquired in the United States--major pathogens. Emerg Infect Dis.

[8] Alkandari A (2017). Temperature and humidity management of the storage houses of food using data logger. Int J New Comput Archit Appl.

[9] Mercier S, Villeneuve S, Mondor M, Uysal I (2017). Time-temperature management along the food cold chain: a review of recent developments. Compr Rev Food Sci Food Saf.

[10] Ercolini D, Russo F, Nasi A, Ferranti P, Villani F (2009). Mesophilic and psychrotrophic bacteria from meat and their spoilage potential in vitro and in beef. Appl Environ Microbiol.

[11] Gogou E, Derens E, Alvarez G, Taoukis P (2014). Field test monitoring of the food cold chain in European markets. 3rd IIR Conference on Sustainability and the Cold Chain, June 2014, London, France.

[12] Macías-Rodríguez ME, Navarro-Hidalgo V, Linares-Morales JR (2013). Microbiological safety of domestic refrigerators and the dishcloths used to clean them in Guadalajara, Jalisco, Mexico. J Food Prot.

[13] Darchuk EM, Waite-Cusic J, Meunier-Goddik L (2015). Effect of commercial hauling practices and tanker cleaning treatments on raw milk microbiological quality. J Dairy Sci.

[14] Sibomana MS, Ziena LW, Schmidt S, Workneh TS (2017). Influence of transportation conditions and postharvest disinfection treatments on microbiological quality of fresh market tomatoes (cv. Nemo-Netta) in a South African supply chain. J Food Prot.

[15] Gornik SG, Albalat A, Macpherson H, Birkbeck H, Neil DM (2011). The effect of temperature on the bacterial load and microbial composition in Norway lobster (Nephrops norvegicus) tail meat during storage. J Appl Microbiol.

[16] Marklinder IM, Lindblad M, Eriksson LM, Finnson AM, Lindqvist R (2004). Home storage temperatures and consumer handling of refrigerated foods in Sweden. J Food Prot.

[17] Altunatmaz SS, Issa G, Aydin A (2012). Detection of airborne psychrotrophic bacteria and fungi in food storage refrigerators. Braz J Microbiol.

[18] Aydin A, Colak H, Ciftcioglu G, Ugur M (2006). Changes in microbiological properties of boneless beef in a one-year study. Arch für Lebensmittelhyg.

[19] Estrada-Flores S, Eddy A (2006). Thermal performance indicators for refrigerated road vehicles. Int J Refrigeration.

[20] Pereira VF, Doria ECB, Júnior BC, Filho LCN, Júnior VS (2010). Evaluation of temperatures in a refrigerated container for chilled and frozen food transport. Food Sci Technol.

[21] Parfitt J, Barthel M, Macnaughton S (2010). Food waste within food supply chains: quantification and potential for change to 2050. Philos Trans R Soc Lond B Biol Sci.

[22] Novaes AG, Lima JR, de Carvalho CC, Bez ET (2015). Thermal performance of refrigerated vehicles in the distribution of perishable food. Pesqui Oper.

[23] Mamot M, Khairuddin NSA (2018). Measuring hand hygiene practice: comparison between self-reported and direct observation among food truck vendors in Klang Valley, Malaysia. Int J Res Pharm Sci.

